# Fluorescent Quinoline-Based Supramolecular Gel for Selective and Ratiometric Sensing Zinc Ion with Multi-Modes

**DOI:** 10.3390/gels8100605

**Published:** 2022-09-21

**Authors:** Qingqing Han, Qingqing Wang, Aiping Gao, Xuefei Ge, Rong Wan, Xinhua Cao

**Affiliations:** 1 Henan Province Key Laboratory of Utilization of Non-Metallic Mineral in the South of Henan Green Catalysis, Synthesis Key Laboratory of Xinyang City, College of Chemistry and Chemical Engineering, Xinyang Normal University, Xinyang 464000, China; 2 Department of Chemistry, Fudan University, Shanghai 200438, China

**Keywords:** quinolone, self-assembly, gel, sensor, ratiometric detection

## Abstract

A gelator **1** containing functional quinoline and Schiff base groups that could form organogels in DMF, DMSO, acetone, ethanol and 1,4-dioxane was designed and synthesized. The self-assembly process of geator **1** was characterized by field emission scanning electron microscopy (FESEM), UV-vis absorption spectroscopy, fluorescence emission spectroscopy, Fourier transform infrared spectroscopy(FTIR), X-ray powder diffraction (XRD) and water contact angle. Under non-covalent interactions, gelator **1** self-assembled into microbelts and nanofiber structures with different surface wettability. Weak fluorescence was emitted from the solution and gel state of **1**. Interestingly, gelator **1** exhibited good selectivity and sensitivity towards Zn^2+^ in solution and gel states along with its emission enhancement and change. The emission intensity at 423 nm of solution **1** in 1,4-dioxane was slightly enhanced, and a new emission peak appeared at 545 nm along with its intensity sequentially strengthened in the titration process. The obvious ratiometric detection process was presented with a limit of detection (LOD) of 5.51 μM. The detection mechanism was revealed by a theoretical calculation and NMR titration experiment, which was that Zn^2+^ induced the transition from trans- to cis- of molecule **1** and further coordinated with **1**. This study will introduce a new method for the construction of functional self-assembly gel sensors for the detection of Zn^2+^.

## 1. Introduction

In the last few decades, supramolecular chemistry has experienced great development due to its wide application in many fields [1,2,3,4,5]. As a representative self-assembly material, low molecular weight gels (LMWGs) have attracted extensive attention because of their extensive applications including drug delivery [6], light-harvesting [7], sensors [8], materials [9], environmental science [10], biomimetic materials [11], catalysis [12], chiral [13], and so on [14,15,16]. In general, a gelator was self-assembled into a three-dimensional network structure with the coating ability towards solvent molecules and further formed gel under the driving force of noncovalent interaction between gelator and gelator, gelator and solvent, such as intermolecular hydrogen bonds, van der Waals forces, π-π stacking, hydrophobicity and electrostatic forces [17,18,19,20]. Owing to the weakness and reversibility of noncovalent interaction, gels exhibited a distinguished reversible dynamic change, so they can be used as intelligent soft materials to respond to external stimuli, such as light, heat, ultrasound, magnetism, pH and chemical substances [21,22,23,24]. We have reported some multi-functional self-assembly systems that were used to selectively detect metal ions, volatile acids or organic amines in a variety of modes [25,26].

**Scheme 1:**
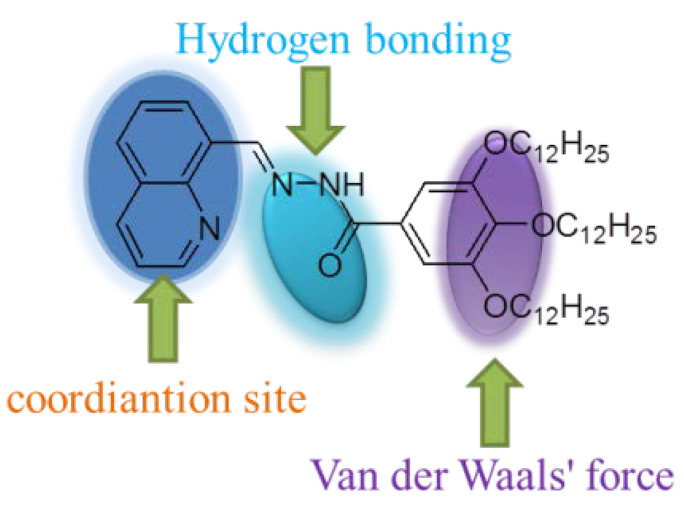
Molecule structure of gelator **1**.

As the second largest trace element in the human body after iron ion, zinc ion plays a key role in human life activities. For example, Zinc ion (Zn^2+^) is involved in the synthesis of various enzymes in the body [27]; disorder of zinc ions (Zn^2+^) in organisms is directly related to a number of diseases, such as immune system disorders, diabetes, epilepsy, Alzheimer’s disease and so on [28,29,30,31]. Therefore, the development of a rapid and sensitive detection method for Zn^2+^ is one of the most popular research hotspots. Fluorescence detection technology has been widely used in zinc ion detection because of its advantages such as good selectivity, high sensitivity and real time performance [32,33,34]. Many different materials have been used to construct fluorescent sensors, such as organic compounds [35], Polyoxometalates [36,37], metal clusters [38], Metal-organic frameworks [39,40,41,42], Covalent Organic Frameworks [43], self-assembly systems [44], semiconductor materials [45,46,47,48,49,50,51,52,53,54], and so on [55]. Compared with other fluorescent sensors, supramolecular self-assembly system sensors exhibit unique advantages including multi-modes (solution, gel and xerogel) and multi-expressive signals (color, fluorescence and gel state) based on the dynamic and reversible nature of their supramolecular noncovalent interactions [56,57]. In this study, a fluorescent gelator (**1**) containing quinolone and schiff base was designed and synthesized (Scheme 1). Nitrogen-atoms in quinolone and Schiff base groups of gelator **1** posess good coordination ability toward metal ions, indicating that some specific metal ions could be selectively detected by this system. As we expected, gelator **1** could selectively detect Zn^2+^ in solution and gel state using a ratiometric mode. This study will introduce a new way of constructing a supramolecular self-assembly fluorescent sensor.

## 2. Results and Discussion

**Figure 1:**
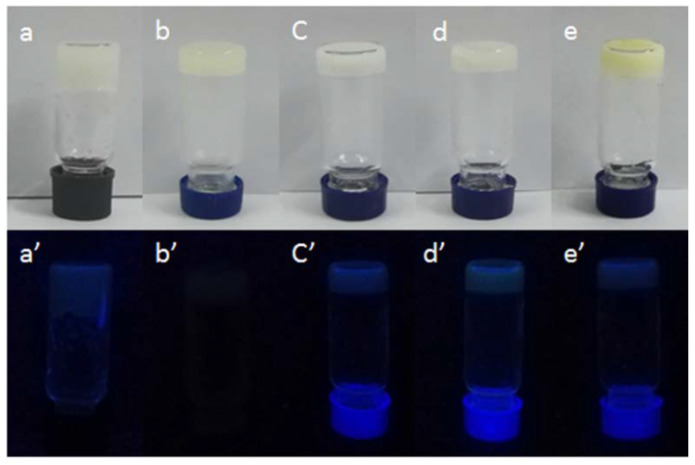
Imagines of gel **1** formed in different solvents: (**a**,**a’**) DMF, (**b**,**b’**) DMSO, (**C**,**C’**) acetone, (**d**,**d’**) 1,4-dioxane, (**e**,**e’**) ethanol. The upper and lower were under daylight and 365 nm light, respectively.

The synthesis and characterization data of gelator **1** are given in the supporting information. In order to explore the gelation ability of gelator **1**, 12 common solvents with different polarity were selected, and the results are demonstrated in Table 1. Gelator **1** could form stable gels in ethanol, acetone, 1,4-dioxane, DMSO and DMF with the critical gel concentrations (CGC) of 8.33, 8.33, 8.33, 12.5 and 8.33 mg mL^−1^, respectively. Sediment was observed in hexane, methanol, acetonitrile, ethyl acetate and petroleum ether in the same condition. On the contrary, solution **1** was obtained in toluene and THF. As shown in Figure 1, gels formed in DMF, DMSO and ethanol were pale yellow and opaque, then gels formed in acetone, 1,4-dioxane were white. Under a portable ultraviolet lamp, gel formed in DMSO could not emit light, while gels formed in other solvents could emit weak blue light.

**Figure 2:**
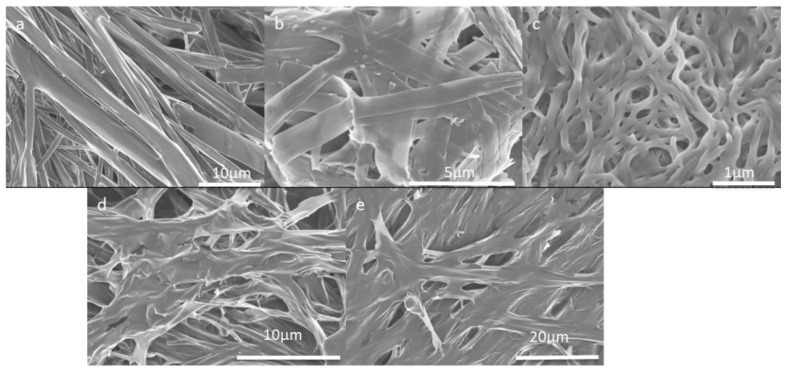
The FESEM imagines of xerogels **1** from different solvents: (**a**) DMF, (**b**) DMSO, (**c**) ethanol, (**d**) 1,4-dioxane, (**e**) acetone.

It is well known that a gelator can self-assemble into different morphologies under the driving force of non-covalent bonding. Field emission scanning electron microscopy (FESEM) was used to investigate the self-assembly structure of **1** in different gels. Gels **1** was diluted with the corresponding solvents, and then evenly dispersed on the mica sheets and finally dried by freeze-drying technology. As shown in Figure 2a,b, **1** was self-assembled into microbelts with the length of dozens of microns and the width of about 2–3 μm in gels from DMF and DMSO. It was quite different that a more compact porous three-dimensional structure was constructed by microfiber with the length of several microns and the width of about 1μm in gel **1** from ethanol (Figure 2c). Irregular microbelts were formed in gels **1** from 1,4-dioxane and acetone (Figure 2d,e). This result demonstrated that solvent molecules could affect the self-assembly process of compound **1**.

**Figure 3:**
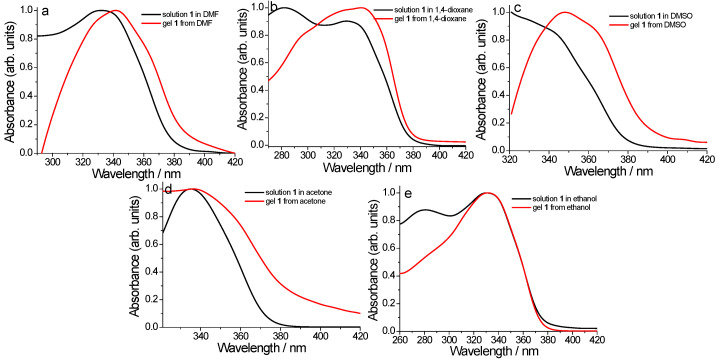
UV-vis absorbance spectra of compound **1** in solution and gel states. (**a**) DMF, (**b**) DMSO, (**c**) 1,4-dioxane, (**d**) acetone, (**e**) ethanol. The concentrations of solution and gel were 10^−5^ M and at its corresponding CGC, respectively.

UV-vis absorption spectra can provide important information about the self-assembly process, especially the method of molecular packing. UV-Vis absorption spectra of compound **1** in solution and gel state in different solvents were carried out and are shown in Figure 3. As shown in Figure 3a, UV-vis absorption bands of compound **1** in DMF was at 332 nm, which was red-shifted to 342 nm in the gel state, indicating J-type aggregation was employed in the gel system [58]. UV-vis spectrum of solution **1** in 1,4-dioxane exhibited two absorption bands at 282 and 329 nm, which were merged into one band at 340 nm for gel state (Figure 3b). A similar result was observed in ethanol, whose two absorption bands at 279 and 329 nm were combined to a single peak at 329 nm (Figure 3e). The UV-vis absorption band of solution **1** in acetone was at 335 nm, which did not show any shift for the gel state (Figure 3d). UV-vis spectrum of gel **1** from DMSO was shifted to 349 nm from 340 nm of solution **1** in DMSO (Figure 3c). From the above experiment, it showed there were different self-assembly modes in gels **1** from different solvents.

**Figure 4:**
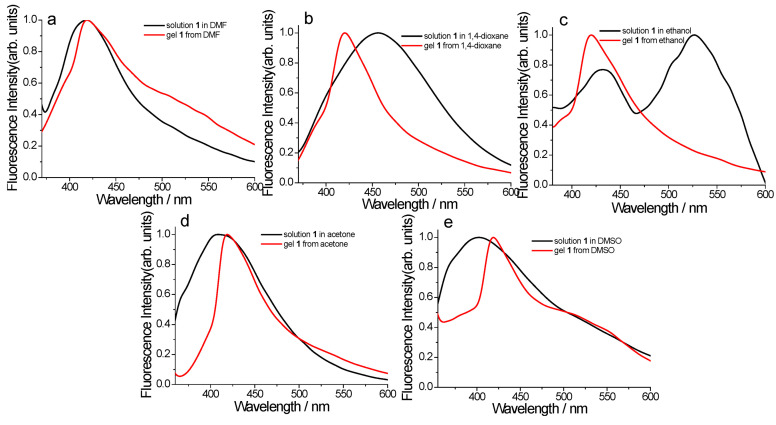
Fluorescence emission spectra of **1** in solution and gel state. (**a**) DMF, (**b**) 1,4-dioxane, (**c**) ethanol, (**d**) acetone, (**e**) DMSO, respectively. The concentrations of solutions were 10^−5^ M and gels were at corresponding CGC.

Fluorescence emission behavior plays an important role in photo-functional materials. Fluorescence emission spectra of compound **1** in solution and gel states in five solvents were carried out (Figure 4). The solution of compound **1** in DMF had a maximum emission wavelength at 417 nm and was slightly red-shifted to 419 nm in gel state (Figure 4a). This was different than the maximum emission peak of gel **1** in 1; 4-dioxane was shifted to 419 nm from 458 nm of its homologous solution with a blue-shift of 39 nm, which exhibited the TICT property in DMSO (Figure 4b) [59]. As shown in Figure 4c, the two emission peaks of solution **1** in ethanol were at 431 and 526 nm, and they were blue-shifted to 420 nm for the gel state. For solution **1** in acetone and DMSO, it had maximum emission wavelengths at 409 and 402 nm, respectively, then were all red-shifted to 419 nm (Figure 4d,e). From this experiment, it was concluded that the solvent not only affected self-assembly mode, but also changed their emission.

**Figure 5:**
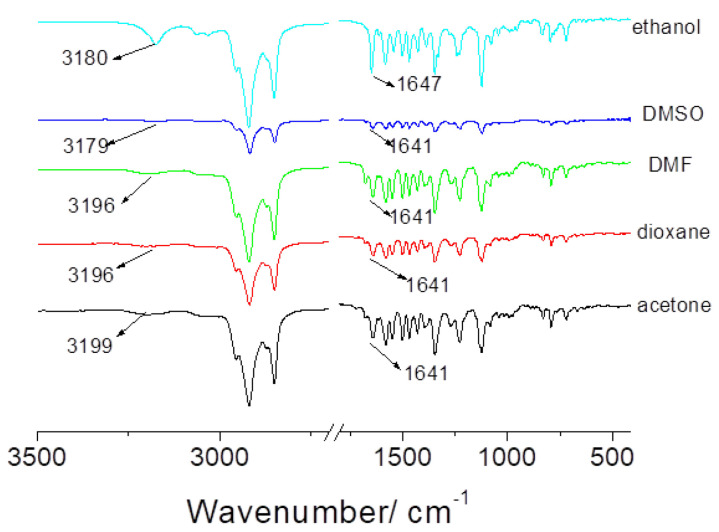
FT-IR spectra of xerogel **1** from different solvents.

Hydrogen bonds are one of the main driving forces for supramolecular self-assembly systems. Fourier transform spectroscopy (FT-IR) can provide important information about hydrogen bond interactions in supramolecular self-assembly systems. FT-IR spectra of xerogel **1** from different solvents were investigated and are shown in Figure 5. The stretching vibration peaks of the N-H band were at 3180, 3179, 3196, 3199 and 3196 cm^−1^ for xerogels **1** from ethanol, DMSO, DMF, acetone and 1,4-dioxane, respectively. The stretching vibration peaks of C=O band were at 1647, 1641, 1641, 1641 and 1641 cm^−1^ for xerogels 1 from different solvents, which precisely indicated the presence of a hydrogen bond in this self-assembly system [60].

**Figure 6:**
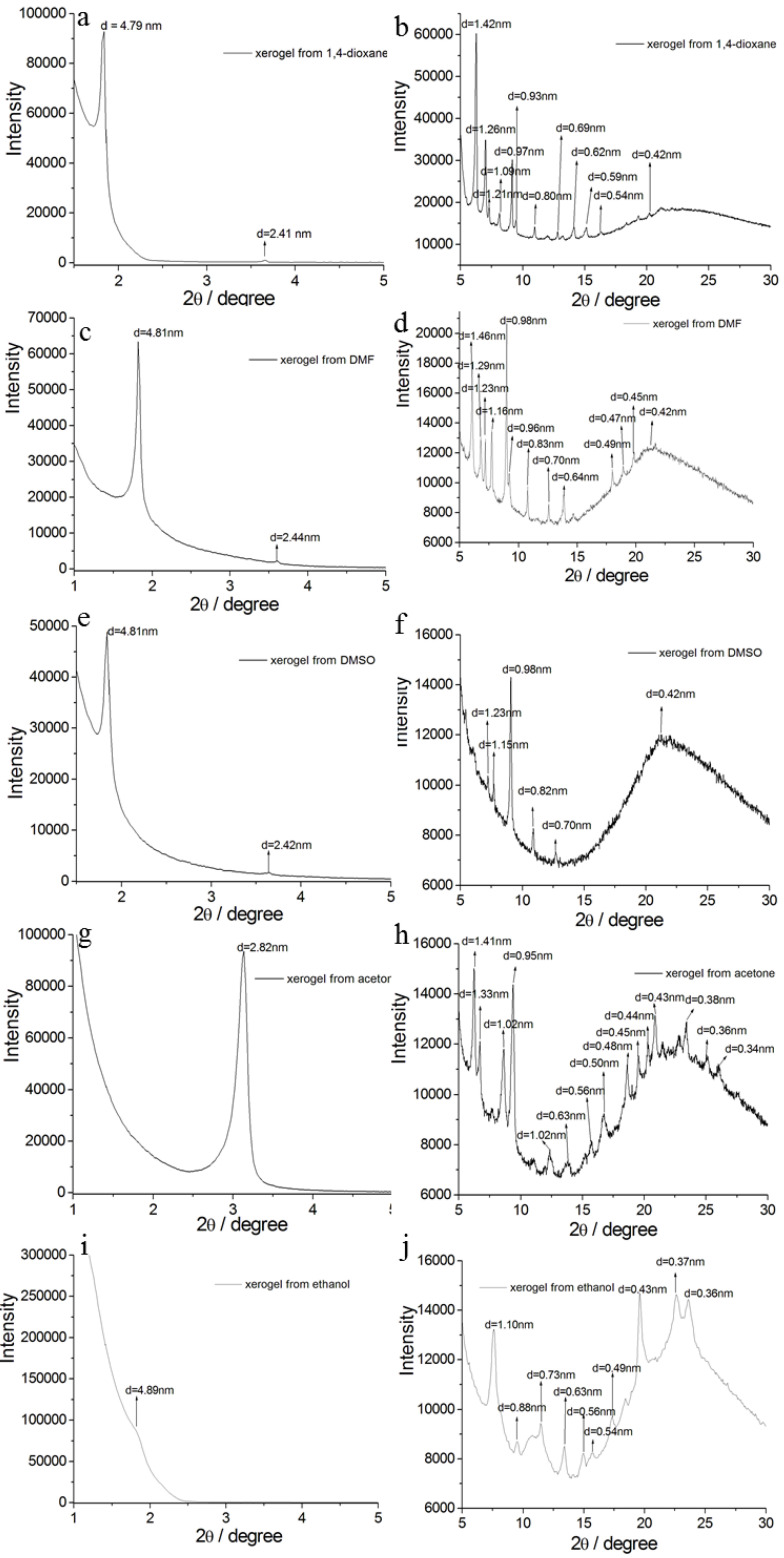
XRD patterns of xerogel **1** formed in different solvents. (**a**,**b**) dioxane, (**c**,**d**) DMF, (**e**,**f**) DMSO, (**g**,**h**), acetone, (**i**,**j**) ethanol.

To some extent, self-assembly information of supramolecular gels, especially the aggregation pattern could be investigated by XRD experiments. XRD patterns of xerogel **1** from 1,4-dioxane showed a series of diffraction peaks with *d*-space values of 4.79, 2.41, 1.42, 1.26, 1.21, 1.08, 0.97, 0.93, 0.80, 0.69, 0.62, 0.59, 0.54 and 0.42 nm (Figure 6a,b). Among the series of diffraction peaks, the d-space values of 4.79, 2.41 and 1.21 nm were in the ratio of 1:1/2:1/4, indicating a layered structure with the spacing of 4.79 nm [61]. The distance between two layers of 4.79 nm was larger than the length of single molecule, but smaller than that of two molecules, which showed that the self-assembly was in a twisty two-molecular mode. A similar packing pattern also existed in gels from DMF and DMSO (Figure 6c–f). However, for gels **1** formed in acetone and ethanol, there is no such proportion relation of *d*-space values, indicating that the accumulation mode in acetone and ethanol was different from that of gels **1** in other three solvents (Figure 6g–j). At the same time, the diffraction peaks with the d-space of 0.38 nm and 0.37 nm in X-ray diffraction patterns of xerogels **1** from acetone and ethanol were characteristic of a π-π stacking distance, which further demonstrated that there existed extensive π-π interactions between quinoline rings of **1** (Figure 6h,j) [62].

**Figure 7:**
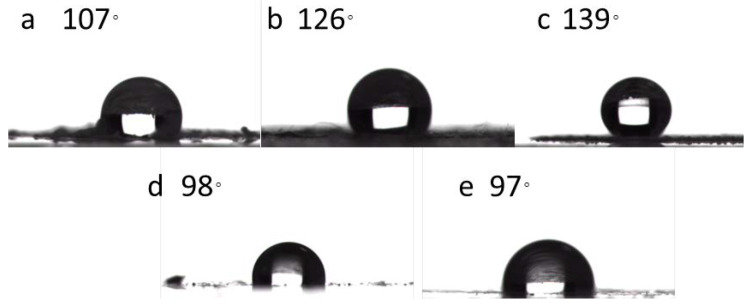
Water contact angle experiment of xerogel films **1** from different solvents: (**a**) acetone, (**b**) 1,4-dioxane, (**c**) ethanol, (**d**) DMF and (**e**) DMSO.

Surface wettability was an important property for surface functional materials, which were applied in many fields, such as industry, agriculture and food industry. For self-assembled systems, molecules could self-assemble into nanostructures with different roughness in different solvents, even if the same molecule and different wettability surfaces can be conveniently constructed [63]. The surface wettability of material was measured by the measurement of the water contact angle. The smaller the contact angle is, the larger the hydrophilicity is. If not, it is the opposite. The surface wettability of xerogels **1** from different solvents are studied and exhibited in Figure 7. Xerogels **1** all exhibited different hydrophobicity. Xerogel **1** from ethanol showed the largest hydrophobic with a contact angle of 139°. On the contrary, xerogel **1** from DMSO owned the largest hydrophilcity with a contact angle of 97°. The other xerogels **1** from acetone, 1,4-dioxane and DMF all had certain hydrophobicity with the contact angles of 107°, 126°, 139° and 98°, respectively. From SEM in Figure 2, the difference in hydrophobicity was possible due to their difference in surface roughness, especially for the large roughness in xerogel from ethanol and the small roughness in xerogel from DMSO, inducing the large and small hydrophobicity, respectively.

**Figure 8:**
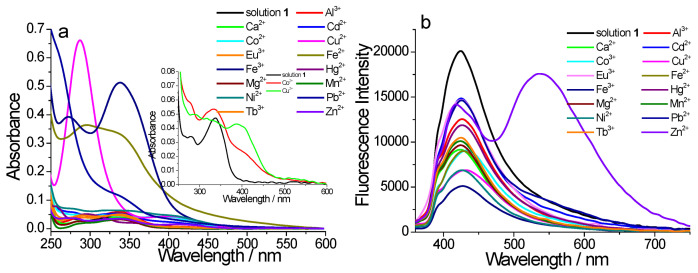
UV-vis absorption (**a**) and fluorescence spectra (**b**) changes of compound **1** in 1,4-dioxane (10^−5^ M) solution adding 10 eq. of different metal ions.

**Figure 9:**
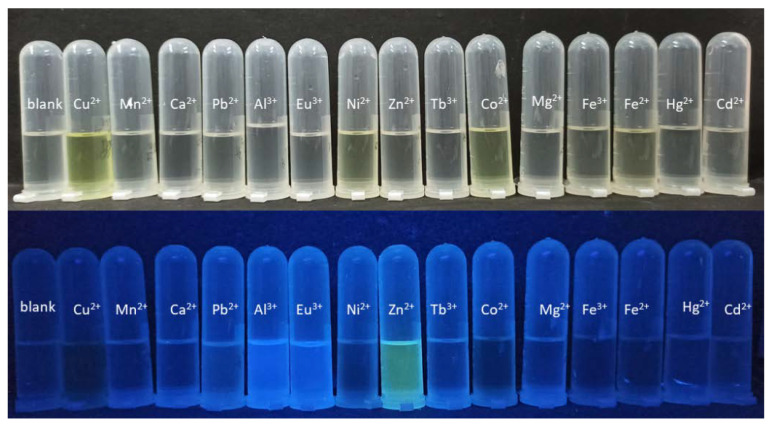
The images of solution **1** in dioxane with 10 eq. different metal ions. The upper and lower were obtained at daylight and under 365 nm.

**Figure 10:**
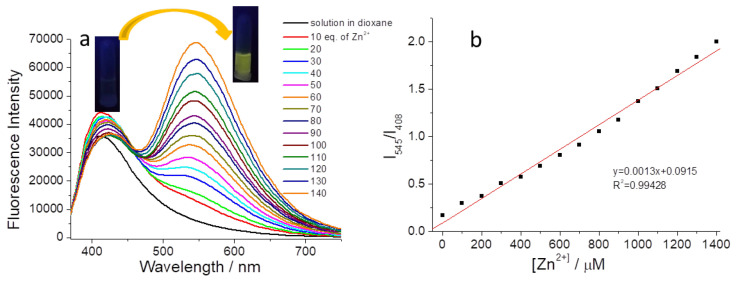
(**a**) The fluorescence titration of solution **1** by Zn^2+^. (**b**) The curves of the fluorescence emission intensity ratio of I_545_/I_408_ of solution **1** versus the concentration of Zn^2+^. The inset figures were images of solution **1** before and after adding Zn^2+^ under 365 nm light lamp.

**Figure 11:**
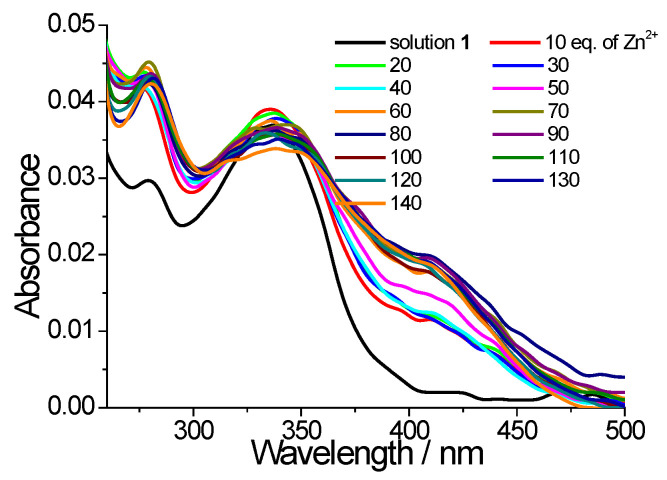
UV-vis absorption spectra change of solution **1** in 1,4-dioxane upon the titration of Zn^2+^.

**Figure 12:**
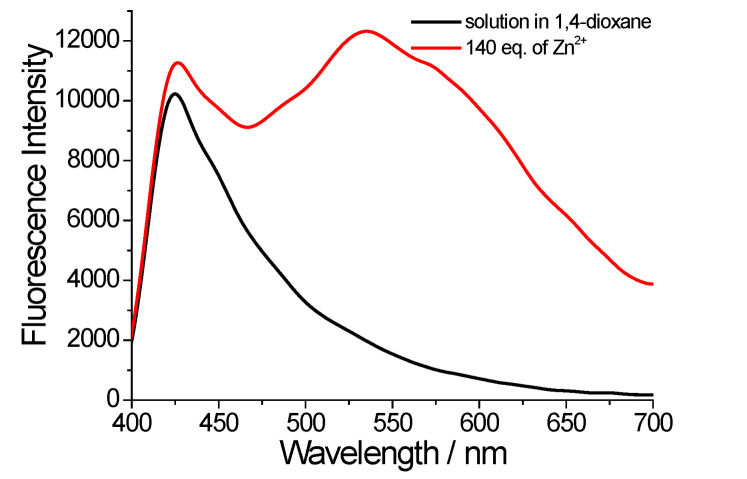
Fluorescence emission spectra change of solution **1** in 1,4-dioxane (kept for three months later) upon the addition of 140 eq. of Zn^2+^.

The structure of gelator **1** contained quinolone and Schiff base groups with three nitrogen-atoms, which could easily coordinate with metal ions and express obvious signals. To investigate the stimulus responsiveness and selectivity of gelator **1** towards different metal ions, 10 eq. of different metal ions (Al^3+^, Ca^2+^, Cd^2+^, Co^2+^,Cu^2+^, Eu^3+^, Fe^2+^, Fe^3+^, Hg^2+^, Mg^2+^, Mn^2+^, Ni^2+^, Pb^2+^, Tb^3+^ and Zn^2+^) were added to solution **1** in 1,4-dioxane and their UV-vis absorption and fluorescence emission spectra were investigated (Figure 8). After the addition of 10 eq. of different metal ions, UV-vis absorption of solution **1** exhibited a different change. Only the addition of Co^2+^ and Cu^2+^ induced the absorbance to increase in the visible region (Figure 8a). Under daylight, the color of solution **1** with Co^2+^ and Cu^2+^ brought obvious yellow from the original colorless (Figure 9). As shown in Figure 8b, the maximum emission wavelength of solution **1** was at 423 nm. With the addition of Zn^2+^ to solution **1**, fluorescence emission intensity at 423 nm was decreased and a new emission peak appeared at 537 nm. Under the irradiation of 365 nm light, the images of solutions **1** with different metal ions provided a solid proof via their emission light change. Emission of solution **1** with Zn^2+^ changed into yellow green light from blue light of the original solution **1** (Figure 9). After the addition of other metal ions, the maximum fluorescence intensity was only decreased with different degrees and no obvious shift. The emission change in solution **1** with other metal ions was not obvious because of the original weak fluorescence of the blank solution (Figure 8b). This result clearly indicated that gelator **1** could be used as a ratiometric probe for the selective detection of Zn^2+^. In order to further explore the detection ability of gelator **1** towards Zn^2+^, the fluorescent titration experiment of solution **1** in 1,4-dioxane by Zn^2+^ was carried out and is shown in Figure 10. With the addition of Zn^2+^ to solution **1**, the emission at 408 nm had a slight change, and a new emission peak appeared and gradually red-shifted to 545 nm (Figure 10a). The linear relationship between the concentration of Zn^2+^ and the fluorescence intensity ratio of I_545_/I_408_ produced the linear equation of y = 0.0013x + 0.0915 with linearly dependent coefficient R^2^ = 0.99428 (Figure 10b). The LOD of solution **1** toward Zn^2+^ was calculated as 5.51 μM by 3*σ*/k, where σ is the standard deviation of the blank solution and k is the slope of the calibration curve [64]. Although, the titration process of Zn^2+^ of solution **1** induced some fluctuation about its absorbance in the visible region, the slight upthrow did not result in a great change in color (Figure 11). As shown in Figure 12, the same fluorescence change was observed on solution **1** in 1,4-dioxane after the addition of 140 eq. of Zn^2+^, which indicated gelator **1** had the ability of long-term measurement. Comparing the result with the previous literature (Table S1), it was found that the LOD of Zn^2+^ in this work was at a moderate level [65,66,67,68,69,70].

**Figure 13:**
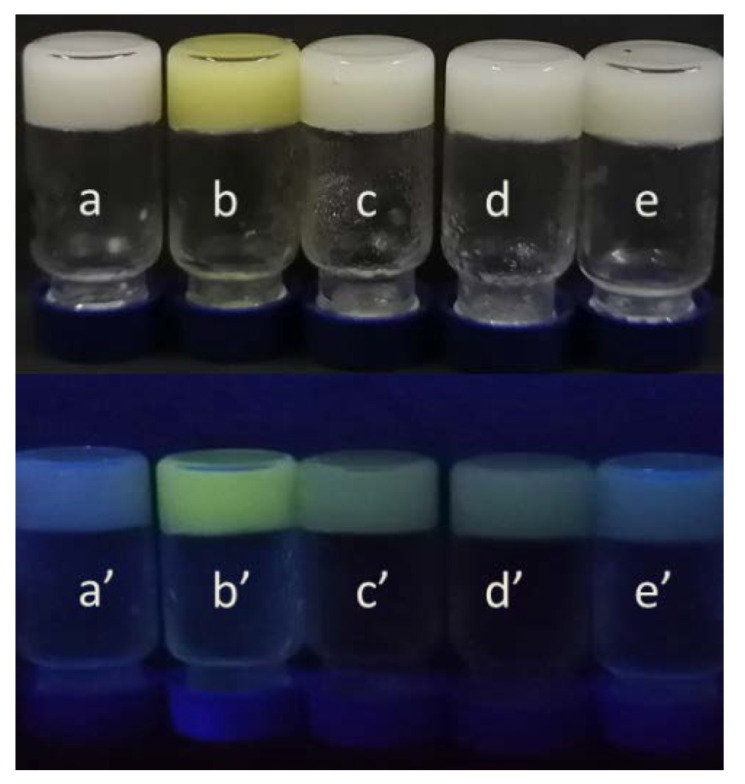
Images of gel **1** from 1,4-dioxane with the addition of Zn^2+^ at different concentrations. (**a**,**a’**) for the free gel, (**b**,**b’**) for 10^−3^ M, (**c**,**c’**) for 10^−4^ M, (**d**,**d’**) for 10^−5^ M, (**e**,**e’**) for 10^−6^ M. The upper and lower were under daylight and UV light (365 nm).

It is well known that the gel system is a three-dimensional network with a large contact area for analytes. Meanwhile, the nature of gel between solid and liquid endows the convenience of analytes entering into gel [71]. Therefore, gel **1** from 1,4-dioxane was also employed as a sensor for the selective detection of metal ions. As shown in Figure S1, fluorescence intensity at 408 nm of gel **1** was only weakened with different degrees under the addition of 1.0 eq. of Al^3+^, Cd^2+^, Co^2+^, Eu^3+^, Fe^2+^, Fe^3+^, Hg^2+^, Mg^2+^, Mn^2+^, Ni^2+^, Pb^2+^, Tb^3+^, and did not show any shift. For the addition of Ca^2+^ or Cu^2+^, the emission intensity of gel **1** was greatly decreased. However, gel **1** did not handily detect Ca^2+^ or Cu^2+^ due to its fundamental weak fluorescence emission and little contrast difference. Interestingly, the addition of Zn^2+^ to gel also brought great change in the fluorescence emission, and a new emission peak appeared at 550 nm with a large width. At the same time, the color and gel state was significantly changed under the addition the aforementioned metal ions. As shown in Figure S2, it had no change under daylight and UV light (365 nm) after the addition of Eu^3+^, Tb^3+^, Mn^2+^ and Mg^2+^. For the addition of Hg^2+^, Co^2+^, Ni^2+^ and Fe^3+^, they induced the gel–sol transition. Additionally, there were varying degrees of color visible to the naked eye. However, the changes in images under 365 nm after adding the mentioned ions were not obvious. With the addition of Cd^2+^, Pb^2+^, Al^3+^, Ca^2+^, Fe^2+^ and Cu^2+^, the colors of the gels **1** were changed to pale yellow, yellow or brown, but the images under 365nm did not show evident change. While the color of the gel with the addition of Zn^2+^ distinctly changed to yellow, yellow green light was emitted from gel under 365 nm light. The result demonstrated that the gel **1** from 1,4-dioxane could also selectively detect Zn^2+^. In order to explore the detection ability of gel **1** toward Zn^2+^, different concentrations of aqueous solution of Zn^2+^ were added onto the surface of gel **1**. As shown in Figure 13, the minimum concentration of Zn^2+^ that could be observed by the naked eye was 10^−5^ M under the UV lamps at 365 nm.

**Figure 14:**
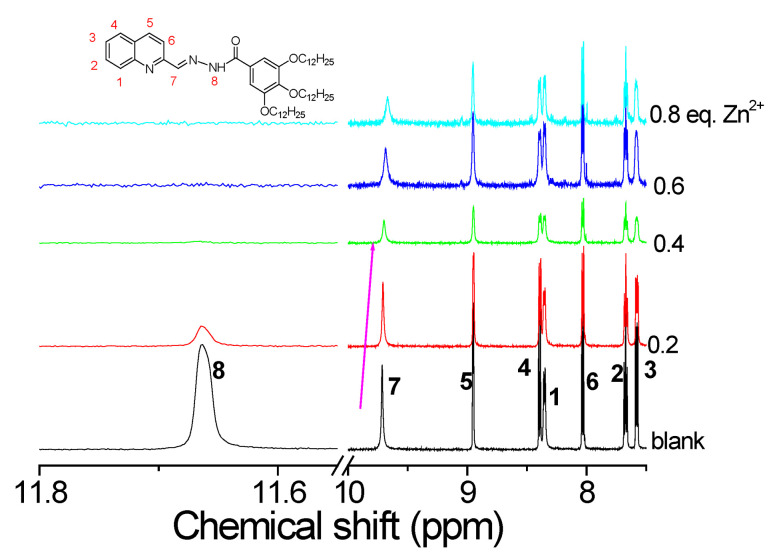
^1^HNMR titration of solution **1** in DMSO-*d_6_* by Zn^2+^.

**Figure 15:**
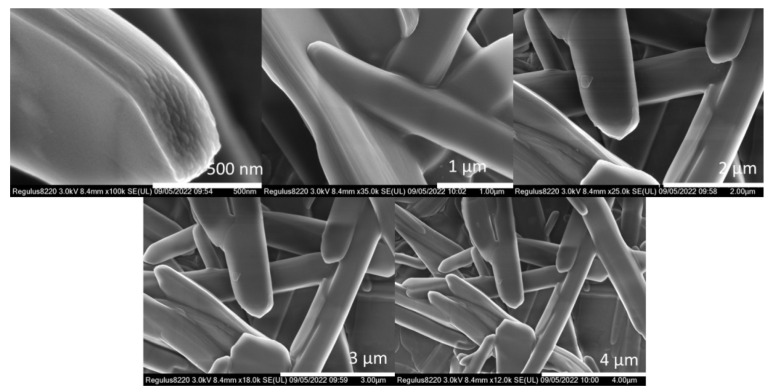
SEM images of gel **1** from 1,4-dioxane after the addition of 1.0 eq. of Zn^2+^.

**Figure 16:**
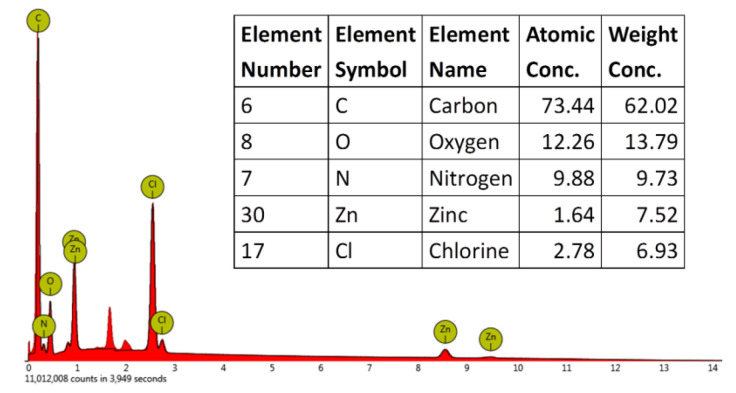
EDX spectra of xerogel **1** with addition of 1.0 eq. of Zn^2+^.

In order to understand the detection mechanism of **1** towards Zn^2+^, theoretical investigations of compound 1 and its Zn^2+^ complexes were performed. The geometry optimizations of simplified compound **1** (trans-and cis-) and complex **1** were carried out using the hybrid TD-B3LYP/6-31G* density functional theory method (DFT). The HOMO and LUMOs of compound **1** and the complexes are listed in Table 2. For simplified compound **1** (trans-), the HOMO and LUMO were all located in the quinolone group, and the HOMO and LUMO of compound **1** (trans-) were −5.64 eV and −1.68 eV, respectively. For simplified compound **1** (cis-), the HOMO primarily resided on the quinolone and benzene ring groups, but the LUMO was only located on the quinolone group. The HOMO and LUMO of compound **1** were −5.75 eV and −1.57 eV, respectively. After the formation of complexes, the HOMO and LUMO all resided in the quinolone group. The HOMO and LUMO of the complex were −5.01 eV and −2.30 eV, respectively. The energy gap between HOMO and LUMO of the complex was 2.71 eV, which was significantly smaller than and 3.96 eV of compound **1** (trans-) and 4.18 eV of compound **1** (cis-), adequately indicating the fluorescence change before and after the addition of Zn^2+^, and the trans-compound **1** was transformed into the cis-compound under the driving force of the coordination of Zn^2+^. To further verify the detailed coordination between compound **1** and Zn^2+^, the NMR titration experiment of solution **1** in DMSO-*d_6_* by Zn^2+^ was conducted and the result is shown in Figure 9. The proton signal of H8 assigned to the amide group at 11.66 ppm was gradually decreased, and ultimately disappeared after the addition of 0.4 eq. of Zn^2+^, which showed the N atom of the amide group participated in the coordination and took place during deprotonation (Figure 14). At the same time, the proton signal of H7 belonging to schiff base was shifted up-field from 9.714 to 9.669 ppm due to the coordination between quinolone and Zn^2+^. The above experiments clearly illustrated the detection mechanism and the coordination mode. In addition, the self-assembly structure of gel **1** after the addition of 1.0 eq. of Zn^2+^ was investigated by SEM for the confirmation of the coordination between **1** and Zn^2+^. The pure zinc ion film of SEM images with the scale bars of 500 nm, 1 µm, 2 µm, 3 µm and 4 µm and EDX spectra were obtained. As shown in Figure 15 and Figure 16, it was not found that the Zinc nanoparticle was formed after the addition of Zn^2+^ and the fiber structure was still maintained. EDX spectra showed that the content of Zn^2+^ was about 7.52%, which was very close to the theoretical value of 6.62%.

## 3. Conclusions

In summary, we designed a fluorescent supramolecular self-assembly system which could form stable gel in five organic solvents and self-assemble into nanofiber or microbelts with different sizes. UV-vis absorption spectroscopy revealed that the *J*-type aggregation mode existed in the self-assembly system. Fluorescence emission and FT-IR spectra indicated that π-π stacking and hydrogen bonds were the main driving force for the formation of gel. Xerogel films from different solvents all showed a series of hydrophobic properties with contact angles of 97°–139°. More interestingly, compound **1** had sensitive and selective response ability towards Zn^2+^ in a ratiometric mode. The blue fluorescence light at 408 nm emitted from compound **1** in solution and gel was changed into yellow light at 545 nm upon the addition of Zn^2+^. The emission change in the detection process was due to its change in molecule structure and the coordination effect. However, the noncovalent interaction was still strong enough to support its gel state, even with the addition of Zn^2+^. This work not only provides a visible and fast method for the detection of Zn^2+^, but also broadens the application of supramolecular self-assembly gel.

## 4. Experimental Section

### 4.1. Reagents and Solvents

Quinoline-8-carbaldehyde was purchased from Sinopharm Chemical Reagent Co., Ltd. (Shanghai, China). Methyl gallate were purchased from Shanghai Titan technology Co., Ltd. (Shanghai, China). 1-Bromododecane and hydrazine hydrate were purchased from Shanghai Darui finechemical Co., Ltd. (Shanghai, China). All the reagents and solvents were analytically pure and without further purification.

### 4.2. Gelation Test

Gelator and solvent were put in test bottles with a sealed cap and heated until the solid was completely dissolved. Then, the sample bottle was cooled to 25 °C (room temperature). Qualitatively, gel was successfully prepared if no sample flowed when the vial was inverted at room temperature (inverted method) [72].

### 4.3. Techniques and Instrumentations

^1^HNMR and ^13^CNMR spectrogram were recorded in DMSO-*d*_6_ on a 600 MHz and 150 MHz nuclear magnetic resonance spectrometer from Japan Electronics Co., Ltd. (JNM–ECZ600R/S3) (Tokyo, Japan). High resolution mass spectrometer (HRMS) was recorded on an LTQ-Orbitrap mass spectrometer (Thermo Fisher Technology Co., Ltd., Minato, Tokyo). FESEM images were obtained using the SEM S4800 instrument (Hitachi, Ltd., Tokyo, Japan). Samples were prepared by dropping diluted gel on silicon wafer, freeze-drying and coating with Pt. Powder X-ray diffractions were performed by using a Philips PW3830 (Philips, Ltd., Eindhoven, The Netherlands) with a power of 40 kV at 40 mA (Cu target, λ = 0.1542 nm). UV–vis absorption spectra were recorded on a UV–vis 3900 spectroscope (Hitachi, Ltd., Tokyo, Japan). Fluorescent spectra were obtained on Edinburgh Instruments FLS 1000 (Edinburgh Instruments, Ltd., Livingston, UK). Fourier transform infrared spectroscopy (FT-IR) was measured by a Nexus 470 spectrometer (Thermo Fisher Scientific Co., Ltd.). Water contact angles were performed using the sessile drop method (Dataphysics, OCA 20). The water droplets were introduced using a microsyringe, and images were captured to measure the angle of the liquid-solid interface; all samples were recorded at three different points. The structure of the HOMO and LUMO states of **1** with simplification were determined with the help of theoretical calculations in the framework of density functional theory (DFT) calculations, at the level of B3LYP/6-31G* in a suite of the Gaussian 09 programs [73].

## Figures and Tables

**Table 1:** Gelation ability of gelator **1** in different solvents.

Solvents	1	Solvents	1
hexane	P	1,4-dioxane	G(8.33)
toluene	S	ethyl acetate	P
methanol	P	petroleum ether	P
ethanol	G(8.33)	DMSO	G(12.5)
acetonitrile	P	DMF	(8.33)
acetone	G(8.33)	THF	S

P = precipitate; S = soluble; G = gel. The values in parentheses is the Critical gel concentration (CGC, mg mL^−1^).

**Table 2:** Structures of simplified compound **1**, complex and their corresponding the frontier orbitals.

Compound	HOMO (eV)	LUMO (eV)
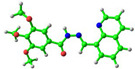 simplified **1** (trans-)	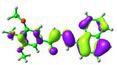 −5.64 eV	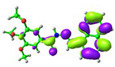 −1.68 eV
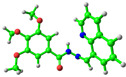 simplified **1** (cis-)	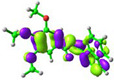 −5.75 eV	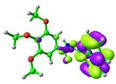 −1.57 eV
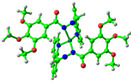 simplified **1** complex	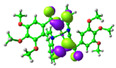 −5.01 eV	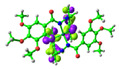 −2.30 eV

## Data Availability

Not applicable.

## Supplementary Materials

The following supporting information can be downloaded at: https://www.mdpi.com/article/10.3390/gels8100605/s1, Scheme S1: The synthesis route of compound 1. Compound **2** (1.0 g, 1.45 mmol), quinoline-8-carbaldehyde (0.23 g, 1.45 mmol) and glacial acetic acid (200 μL) were mixed at ethanol (20 mL). The mixture was refluxed under N_2_ atmosphere for 24 h. After the reaction was complete, it was cooled to room temperature, then white powder was obtained by suction filtration and the filter cake was separated and purified by column layer analysis. (CH_2_Cl_2_:CH_3_OH = 100:1). ^1^HNMR (600 MHz, DMSO-d6): δ 11.72 (s, 1H), 9.75 (s, 1H), 8.99 (d, *J* = 4.1 Hz, 1H), 8.43 (d, *J* = 8.3 Hz, 1H), 8.39 (d, *J* = 6.9 Hz, 1H), 8.07 (d, *J* = 8.3 Hz, 1H), 7.71 (t, *J* = 7.6 Hz, 1H), 7.62 (q, *J* = 4.1 Hz, 1H), 7.30 (s, 2H), 4.10–4.04 (4H), 4.00–3.93 (2H), 1.79–1.74 (m, 4H), 1.69–1.66 (m, 2H), 1.44–1.51 (m, 6H), 1.27–1.38 (m, 48H), 0.86 (t, *J* = 5.9 Hz, 9H). ^13^CNMR (150 MHz, DMSO-d6): δ 198.99, 178.53, 151.75, 150.04, 145.24, 136.27, 129.20, 128.07, 126.32, 121.35, 72.27, 68.79, 30.84, 28.56, 28.33, 28.21, 25.22, 21.57, 13.32. HRMS calculated for C_53_H_85_N_3_NaO_4_, [M + Na]^+^ 850.6438, found 850.6475; Table S1: The molecular structures and LODs of previous reported sensors towards Zn^2+^; Figure S1: The fluorescence change spectra of gel **1** from 1,4-dioxane with the addition of different metal ions; Figure S2: The imagines of gel 1 from 1,4-dioxane with addition of different metal ions (1.0 eq.). The upper and the lower were under daylight and 365 nm light.
