# Supplementary material for: Fluorescent Quinoline-Based Supramolecular Gel for Selective and Ratiometric Sensing Zinc Ion with Multi-Modes
Source: Gels. 2022 Sep 21;8(10):605. doi: 10.3390/gels8100605 (PMC9601706; doi:10.3390/gels8100605)
Supplement: Supplementary file 1 [file gels-08-00605-s001.zip › gels-1816577-supplementary.pdf]

# Fluorescent Quinoline-Based Supramolecular Gel for Selective and Ratiometric Sensing Zinc Ion with Multi-Modes

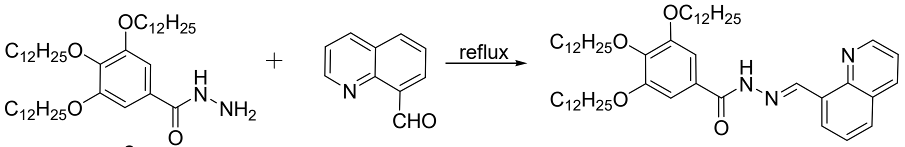


2 1

**Scheme S1.** The synthesis route of compound 1. Compound **2** (1.0g, 1.45mmol), quinoline-8-carbaldehyde (0.23g, 1.45mmol) and glacial acetic acid (200μL) were mixed at ethanol (20ml). The mixture was refluxed under $N_2$ atmosphere for 24h. After the reaction was complete, it was cooled to room temperature, then white powder was obtained by suction filtration and the filter cake was separated and purified by column layer analysis. ($CH_2Cl_2$: $CH_3OH$=100:1). $^1$HNMR (600 MHz, DMSO-d6)：δ 11.72 (s, 1H), 9.75 (s, 1H), 8.99 (d, *J* = 4.1 Hz, 1H), 8.43 (d, *J* = 8.3 Hz, 1H), 8.39 (d, *J* = 6.9 Hz, 1H), 8.07 (d, *J* = 8.3 Hz, 1H), 7.71 (t, *J* = 7.6 Hz, 1H), 7.62 (q, *J* = 4.1 Hz, 1H), 7.30 (s, 2H), 4.10-4.04 (4H), 4.00-3.93 (2H), 1.79-1.74 (m,4H), 1.69-1.66 (m, 2H), 1.44-1.51 (m, 6H), 1.27-1.38 (m, 48H), 0.86 (t, *J* = 5.9 Hz, 9H). $^{13}$CNMR (150 MHz, DMSO-d6)：δ 198.99, 178.53, 151.75, 150.04, 145.24, 136.27, 129.20, 128.07, 126.32, 121.35, 72.27, 68.79, 30.84, 28.56, 28.33, 28.21, 25.22, 21.57, 13.32. HRMS calculated for $C_{53}H_{85}N_3NaO_4$, $[M+Na]^+$ 850.6438, found 850.6475.

**Table S1.** The molecular structures and LODs of previous reported sensors towards $Zn^{2+}$.

| Molecular structure | Switch type | LOD for $Zn^{2+}$ | Ref. |
|---|---|---|---|
| 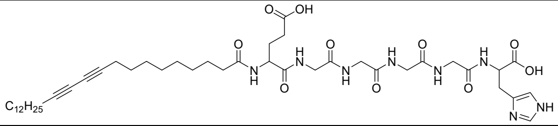 | sol-gel | / | [1] |
| 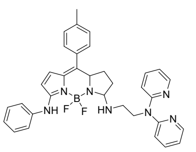 | turn-on | 61.3 nM | [2] |
| 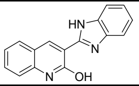 | off-on | 15 μM | [3] |
| 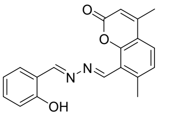 | off–on | 20 μM | [4] |
| 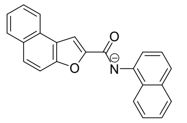 | turn–on | 0.21 μM | [5] |
| 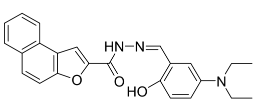 | off-on | 0.13 mM | [6] |

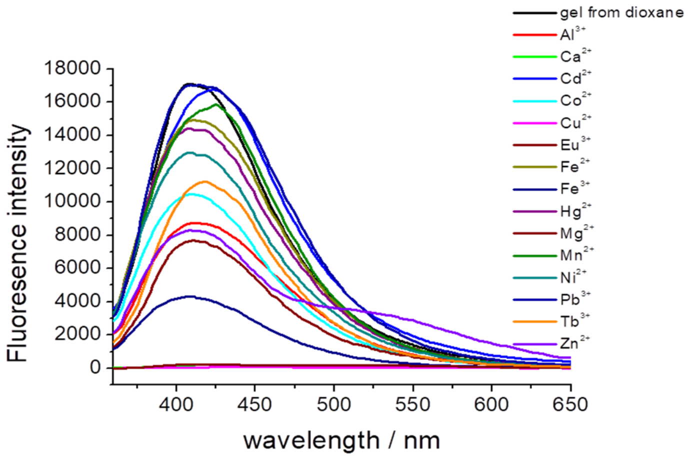


**Figure S1.** The fluorescence change spectra of gel **1** from 1, 4-dioxane with the addition of different metal ions.

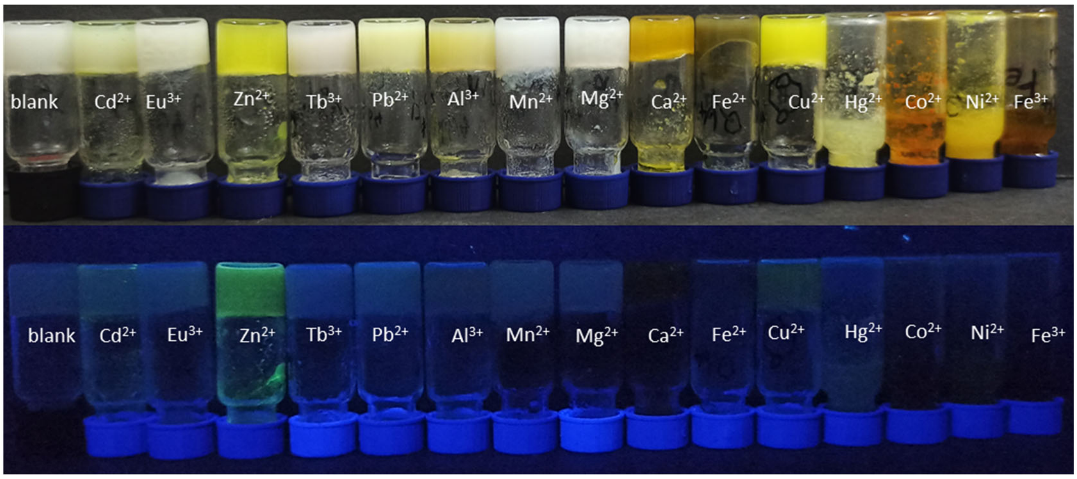


**Figure S2.** The imagines of gel 1 from 1, 4-dioxane with addition of different metal ions (1.0 eq.). The upper and the lower were under daylight and 365 nm light.
